# Mediating Roles of Cognitive Complaints on Relationships between Insomnia, State Anxiety, and Presenteeism in Japanese Adult Workers

**DOI:** 10.3390/ijerph18094516

**Published:** 2021-04-24

**Authors:** Kuniyoshi Toyoshima, Takeshi Inoue, Akiyoshi Shimura, Yoshihiro Uchida, Jiro Masuya, Yota Fujimura, Shinji Higashi, Ichiro Kusumi

**Affiliations:** 1 Department of Psychiatry, Graduate School of Medicine, Hokkaido University, Kita 15, Nishi 7, Sapporo 060-8638, Japan; ikusumi@med.hokudai.ac.jp; 2 Department of Psychiatry, Tokyo Medical University, Shinjuku-ku, Tokyo 160-0023, Japan; tinoue@tokyo-med.ac.jp (T.I.); akiyoshi.shimura@gmail.com (A.S.); yotik2006@yahoo.co.jp (Y.U.); j-masuya@tokyo-med.ac.jp (J.M.); fyota@yahoo.co.jp (Y.F.); shigashi@tokyo-med.ac.jp (S.H.); 3 Department of Psychiatry, Ibaraki Medical Center, Tokyo Medical University, Ami-machi, Inashiki-gun, Ibaraki 300-0395, Japan; 4 Department of Psychiatry, Hachioji Medical Center, Tokyo Medical University, Tokyo 193-0998, Japan

**Keywords:** insomnia, state anxiety, cognitive complaints, presenteeism, mediator

## Abstract

Complaints of cognitive functions (CCFs), defined as subjective cognitive dysfunction, affect social function; additionally, for workers, this condition is an important factor in presenteeism and mediates the effect of depressive symptoms on presenteeism. This study aimed to investigate whether CCFs mediate the relationships among insomnia, state anxiety (SA), and presenteeism. Participants were 471 Japanese adult workers evaluated using the Athens Insomnia Scale, State-Trait Anxiety Inventory (Form Y), Cognitive Complaints in Bipolar Disorder Rating Assessment, and Work Limitations Questionnaire 8 to assess insomnia, SA, CCFs, and presenteeism, respectively. Path analysis was used to evaluate the correlations between variables. CCFs significantly mediated the associations among insomnia, SA, and presenteeism. To address the presenteeism associated with insomnia and SA, it may be useful to assess the mediating roles of CCFs.

## 1. Introduction

Work-related stress affects both physical and mental health in workers and influences the health and performance of organizations [1,2], and approximately 20% of adult workers have mental health issues [3]. Recent research reported a high prevalence of depression, state anxiety (SA), which is defined as a transitory emotional state consisting of feelings of apprehension, nervousness, and physiological sequelae such as an increased heart rate or respiration [4], and insomnia, which is defined as subjective sleep complaints in the general population [5,6], for health care workers during the coronavirus disease 2019 (COVID-19) pandemic [7]. Other studies to address the mental health problems in workers are ongoing.

Cognitive dysfunction has attracted recent attention in the fields of public and occupational health. When assessing cognitive function, objective or subjective cognitive assessments are conducted. Complaints of cognitive functions (CCFs) are defined as subjective cognitive dysfunction, which can be evaluated by subjective cognitive assessments [8,9]. In individuals with psychiatric illness, CCFs correlate with psychosocial functioning [10,11]. In recent years, the clinical importance of CCFs, with or without mental illness, has begun to be recognized. In the general population, CCFs affect social functioning and quality of life [12]. Further, in adult workers, CCFs influence working with limitations because of illness (presenteeism), independent of depressive symptoms [13]. Hence, CCFs are considered as one of the important factors of presenteeism.

Presenteeism is defined as health-related work productivity loss and being present at work while feeling unhealthy [14,15], which worsens the worker’s quality of life [16,17]. In this study, we defined presenteeism as work productivity loss. As factors that affect presenteeism, various health conditions were reported, including insomnia and SA [18]. Recent study suggests that CCFs directly worsen presenteeism, and they are considered to be a key factor of presenteeism [13]. As factors that affect CCFs, childhood parental bonding, affective temperaments, and depressive symptoms have been reported [19]. Recently, the mediating role of CCFs on the relationship between depressive symptoms and presenteeism was reported [13]. However, to our knowledge, the mediating role of CCFs on presenteeism is not fully understood.

Insomnia worsens workers’ mental health and impairs their functioning [20,21]. The relationships of insomnia with occupational accidents, productivity, presenteeism, sick leave, and work-related burnout have been reported [22,23,24,25]. Recent meta-analysis suggested that interventions on insomnia improved workers’ health, which led to improvements in productivity, presenteeism, and job burnout [26].

A recent study reported that insomnia complaints are linked to daytime cognitive performance in individuals with insomnia [27]. A meta-analysis revealed that individuals with insomnia exhibited small to moderate cognitive deficits for working memory, episodic memory and some aspects of executive functioning compared to healthy sleepers [28]. A previous study reported that cognitive deficits for attention and episodic memory were shown in individuals with insomnia [29]. In addition, it was reported that current generalized anxiety disorder moderated the associations between insomnia and memory/concentration problems [30]. However, to our knowledge, the mediating role of SA in the relationship between insomnia and CCFs in workers was not fully understood.

A meta-analysis showed that insomnia increased SA [31], and SA affects cognitive impairment [32]. In addition, CCFs directly affect presenteeism [13]. However, to our knowledge, whether insomnia will be positively related to presenteeism through the mediating influence of SA and, in turn, CCFs is unclear. Hence, we aimed to investigate the mediating influence by evaluating the “insomnia → SA → CCFs → presenteeism” model using path analysis.

Regarding the mediating role of CCFs, several findings have been reported. The impact of affective temperament on functional disability is mediated by CCFs [33]. Furthermore, the impact of depressive symptoms on presenteeism and social function is mediated by CCFs [12,13]. However, to the best of our knowledge, the mediating role of CCFs on the relationships between insomnia, SA, and presenteeism remains unclear. Hence, the hypothesis of this study was that CCFs mediate the associations among insomnia, SA, and presenteeism. CCFs are reported to mediate the relationship between depressive symptoms and presenteeism in the following manner: “depressive symptoms → CCFs → presenteeism” [13]. Further, anxiety reportedly mediates the relationship between sleep satisfaction and cognitive complaints as follows: “sleep satisfaction → anxiety → cognitive complaints” [34]. Hence, we have hypothesized the path model as follows: “insomnia → SA → CCFs → presenteeism”.

## 2. Material and Methods

### 2.1. Participants

The study used a convenience sampling method to recruit 597 adult volunteers in Tokyo between April 2017 and April 2018. The participants were recruited from Tokyo Medical University, and were informed about the study verbally or by using posters. We set the inclusion criteria for volunteers as follows: (a) at least 20 years of age; (b) no serious physical illness; (c) no organic brain damage; and (d) able to provide consent to participate in the present study.

Exclusion criteria were unemployment at the time of assessment and failure to complete the assessment scales. Hence, all participants were workers, and students did not participate in this study. The place of work was not limited to Tokyo Medical University, but there are various places in Tokyo. This study is part of a larger research trial that investigated several scales to evaluate the quality of life in Japanese adults [12]. The research was conducted in accordance with the Declaration of Helsinki [35]. The approval from the Local Ethics Committee of Tokyo Medical University was acquired (approval number: SH3502) and written informed consent was obtained from all participants before starting this study.

### 2.2. Scales

#### 2.2.1. Insomnia

The Athens Insomnia Scale (AIS) was used to evaluate the insomnia severity [36,37]. This scale comprises 8 items, each with a 4-point scale ranging from 0 to 3. This study used the Japanese version of the Athens Insomnia Scale (AIS-J), which was shown to have validity [38]. We calculated the total score, ranging from 0 to 24, and the cut-off score of ≥6 indicated pathologic insomnia [38]. The AIS-J has a two-factor structure: “nocturnal sleep problem” consists of items 1–5, and “daytime dysfunction” consists of items 6–8. High internal consistency was shown; Cronbach’s alpha values for factor 1, factor 2, and the total scores were 0.85, 0.78, and 0.88, respectively [38].

#### 2.2.2. SA

The State-Trait Anxiety Inventory (Form Y) (STAI-Y), which evaluates the degrees of anxiety [39], was used to assess SA in this study. Hence, only the STAI-Y state score was evaluated, which ranged from 20 to 80. This form has 20 items to assess SA that are rated on a 4-point scale. The maximum total score is 80, which indicates extremely severe SA. This study used the Japanese version, which was demonstrated to be valid [40]. High reliability of SA for males (Cronbach’s alpha value = 0.92) and females (Cronbach’s alpha = 0.92) was reported [41].

#### 2.2.3. CCFs

The Cognitive Complaints in Bipolar Disorder Rating Assessment (COBRA) was used to evaluate CCFs [8]. This assessment consists of 16 items related to the performance of daily mental tasks [8,9]. High internal consistency (Cronbach’s alpha = 0.887) for the total score was achieved using the Japanese version of COBRA [9]. Each item is evaluated using a 4-point scale ranging from 0 to 3. The maximum total score is 48, which indicates extremely severe CCFs. Both the current and previous studies used the Japanese version to evaluate CCFs in the general adult population [12].

#### 2.2.4. Presenteeism

The Work Limitations Questionnaire (WLQ), which evaluates health-related working disability [42,43], was used for this study as the Work Limitations Questionnaire 8 (WLQ-8), Japanese version, based on the WLQ-25 [44]. High internal consistency was demonstrated using the Japanese version of WLQ (Cronbach’s alpha ≥ 0.7 for all subscales) [44]. The following choices were applied for each item: “all of the time”; “a great deal of the time”; “some of the time”; “a slight bit of the time”; “none of the time”; and “does not apply to my job” [44]. Using these subscale scores, the work productivity loss score (the percentage of work productivity loss) was calculated to evaluate presenteeism [13]. This score represents the estimated presenteeism, and a higher score indicates severe presenteeism [45].

### 2.3. Statistical Analysis

The strengths of the correlations among the parameters were evaluated using Pearson correlation coefficients [46]. When performing multiple regression analysis, linearity was confirmed by a normal probability plot. Forced entry multiple regression analysis was conducted to evaluate the relationships among insomnia, SA, CCFs, and presenteeism. Path analysis was conducted to evaluate the influences of insomnia, SA, and CCFs on presenteeism. CCFs are known to mediate the relationship between depressive symptoms and presenteeism [13], whereas anxiety is believed to mediate the relationship between sleep satisfaction and cognitive complaints [34]. Thus, we hypothesized the path model as follows, “insomnia → SA → CCFs → presenteeism”. In the path model, we used only observed variables and did not include any latent variables. As the model of this study was a saturation model, the goodness-of-fit index was not used. In the path model, mediation analysis was performed to evaluate the mediating role of CCFs. The standardized path coefficients were calculated to demonstrate the strengths of the effects. We considered the acceptable percentage of missing values was 5%. The missing values were under 5% in this study. Missing values had been removed using the list-wise method. The model of this study was a saturation model; hence, we referred to the following sample size requirements: a minimum sample size of 100 [47,48]. The sample size of this study met the criteria. Statistical analyses were conducted using STATA/MP 16 (StataCorp LLC, College Station, TX, USA), except for the path analysis, which was conducted using Mplus version 8.4 (Muthén & Muthén, Los Angeles, CA, USA). For all analyses in this study, *p* < 0.05 was considered statistically significant.

## 3. Results

### 3.1. Demographic Data

After the exclusion criteria were applied for the 597 recruited volunteers, 471 participants were enrolled and evaluated. Demographic characteristics for the participants in this study are shown in Table 1. The Cronbach’s α of scales was AIS-J (0.83), STAI-Y state (0.71), COBRA (0.91), and WLQ (0.73), respectively.

### 3.2. Relationships between Insomnia, SA, Cognitive Complaints and Presenteeism

Pearson correlation coefficients are shown in Table 2. Significantly moderate positive correlations were observed between insomnia and SA, between insomnia and CCFs, and between CCFs and presenteeism. Significantly weak positive correlations were observed between the other variables.

### 3.3. Multiple Regression Analysis

Results of the multiple regression analysis for presenteeism and CCFs are shown in Table 3. Insomnia, SA, and CCFs were significant positive predictors of presenteeism, while age, married status, education, and smoking were significant negative predictors of presenteeism. Regarding CCFs, insomnia and SA were significant positive predictors of CCFs, while married status was a significant negative predictor of CCFs.

### 3.4. Path Analysis

**Figure 1:**
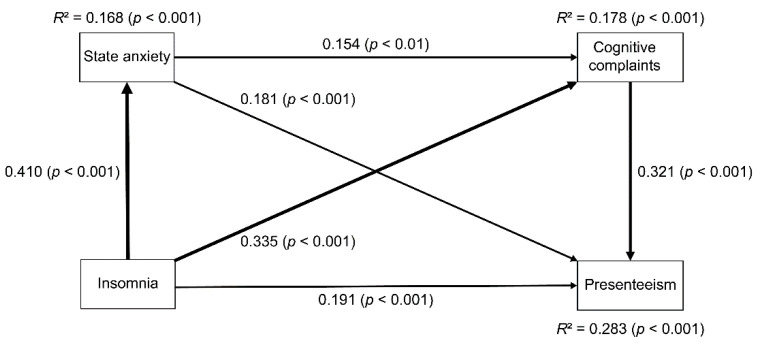
Relationships between insomnia, state anxiety (SA), cognitive complaints, and presenteeism using path analysis in 471 Japanese adult workers. The numbers beside the arrows indicate the direct standardized path coefficients. The width of the line represents the strength of the direct effect. Insomnia represents the AIS-J score, SA represents the STAI-Y state score, cognitive complaints represent the COBRA total score, and presenteeism represents the WLQ work productivity loss score. AIS-J, Athens Insomnia Scale, Japanese version; COBRA, Cognitive Complaints in Bipolar Disorder Rating Assessment; STAI-Y, State-Trait Anxiety Inventory Form Y; *R*^2^, coefficient of determination.

Path analysis was conducted to evaluate the relationships among insomnia, SA, CCFs, and presenteeism (Table 4, Figure 1). In this model, the coefficient of determination of presenteeism was 0.283, which means that the model explained 28.3% of presenteeism in Japanese adult workers. In addition, all the paths were statistically significant (Table 4).

Regarding direct effects, insomnia directly affected SA, CCFs, and presenteeism. SA directly affected CCFs and presenteeism. Furthermore, CCFs directly affected presenteeism.

Regarding indirect effects, insomnia affected CCFs by the pathway of SA. Insomnia affected presenteeism by the pathway of SA, by CCFs, and by both SA and CCFs. SA affected presenteeism by CCFs.

To summarize these findings, the mediating effects of SA and CCFs were shown in the relationship between insomnia, SA, CCFs, and presenteeism.

## 4. Discussion

Our study suggests that insomnia may be positively related to presenteeism through the mediating role of SA and CCFs. Moreover, the mediating roles of CCFs in the relationship between insomnia, SA, and presenteeism have been shown for the first time to our knowledge.

The present study suggests that insomnia may be positively related to SA directly, which is consistent with the previous meta-analysis which suggested that insomnia increased SA [31]. Furthermore, regarding the effect of the length of the period of insomnia, longer insomnia tends to increase SA [31]. Given that insomnia may precipitate and/or maintain anxiety symptoms, the application of sleep interventions is of potential significance for mental health promotion [49]. Hence, considering these, the importance of early intervention for insomnia to address SA in workers was suggested.

This study suggests that insomnia may be positively related to CCFs directly and indirectly via SA. Previous studies reported that insomnia is linked to cognitive performance [27,28,29], and generalized anxiety disorder moderated the associations between insomnia and cognitive function [30]. To our knowledge, the present study is the first to suggest that SA may mediate the relationship between insomnia and CCFs. Hence, considering these, SA could have both a moderating and mediating role in the relationship between insomnia and CCFs. In the future, a longitudinal study should be conducted to clarify the moderating role of SA in the relationship between insomnia and CCFs in adult workers.

Our study suggests that insomnia may be positively related to presenteeism directly. Previous studies reported the negative impact of insomnia on work ability [23,50], which is consistent with the result of this study. Additionally, our model suggests that insomnia may be positively related to presenteeism via SA and CCFs. A previous study reported that depressive symptoms were positively related to presenteeism via CCFs [13]. However, to our knowledge, the mediating role of SA and CCFs in the relationship between insomnia and presenteeism has not been fully understood. Therefore, to the best of our knowledge, this is the first study investigating the mediating role of SA and CCFs in the relationship between insomnia and presenteeism. Our model suggests that insomnia may be positively related to presenteeism via SA and, in turn, CCFs. Previous studies reported that insomnia increased SA [31], and SA affected cognitive impairment [32], and CCFs directly affected presenteeism [13]. To clarify the mediating role of SA and, in turn, CCFs in the relationship between insomnia and presenteeism, a longitudinal study should be conducted in the future.

Regarding the mediating role of CCFs in workers, the results of this study suggest that CCFs mediate the associations of insomnia and SA with presenteeism in Japanese adult workers. To the best knowledge of these authors, this result is a new finding about the mediator role of CCFs in workers. Previous research suggested a mediator role of CCFs for the influence of depressive symptoms on presenteeism [13]. Hence, when considering the influence of insomnia and SA on presenteeism, as in depressive symptoms, the mediating roles of CCFs should be evaluated. In addition, this study suggests a significant indirect effect of insomnia on presenteeism by the pathways of SA and CCFs. Hence, when considering the influence of insomnia on presenteeism by CCFs, the mediator role of SA in the relationship between insomnia and CCFs should be evaluated.

Regarding the mediating role of SA in workers, we suggest that SA mediates the impact of insomnia on CCFs in workers. In adults, affective temperaments and depressive symptoms have been shown to have a mediating role in the relationships between childhood parental bonding and CCFs [19]. Hence, when examining these factors that affect CCFs in workers in future studies, it may also be necessary to evaluate the mediating effects of affective temperaments and depressive symptoms in workers.

Regarding workers’ mental health, research has shown that childhood parental bonding, neuroticism, and resilience affect work-related stress in adult workers [51,52]. Furthermore, sleep disturbance and work-related stress affect presenteeism [53]. A recent study suggests that improving sleep disturbance may reduce a worker’s stress response [54]. Regarding interventions, internet-based cognitive behavioral therapy for insomnia may be a cost effective strategy [55]. In addition, digital cognitive behavioral therapy may be effective to improve insomnia and work productivity loss in workers with insomnia [56]. From the results of the path analysis in the current study, improving insomnia may reduce SA, CCFs, and presenteeism. Furthermore, to address the presenteeism associated with insomnia, it may be effective to intervene not only for the insomnia but also for SA and CCFs simultaneously. One of the promising interventions, Stand More AT Work intervention, could be effective for presenteeism [57]. In particular, considering this study’s findings on the mediating roles of CCFs, it may be necessary to develop interventions that directly target CCFs. Recent research emphasizes the importance of promoting sleep quality to improve CCFs [34]. Hence, our findings on the mediating role of SA in the association between insomnia and CCFs may also contribute to the development of interventions that directly target CCFs.

Regarding the functional disability of workers, CCFs are associated with functional disability not only including work, but also in the context of social and family functioning [12]. Although the present study investigated the relationship between CCFs and work limitations, further research is needed to elucidate the relationships among CCFs and insomnia, SA, and functional disability in workers.

Regarding the management implications, the negative impact of presenteeism is not only with the individual but with the whole organization; the organization’s actions could be decisive in the improvement of workplaces [58]. Furthermore, presenteeism has potential impacts on workers’ health, and managing the worker’s responsibilities is important [59]. With respect to wage loss due to presenteeism, depression, anxiety, and emotional disorders were considered to be the leading causes in Japanese workers [60]. Hence, developing strategies for workplace interventions on increasing work performance and workers’ mental health may need to be conducted, simultaneously. Adequate assessment and developing management tools would contribute to reducing the impact of presenteeism [61]. Hence, our path model may contribute to assessing presenteeism adequately and thereby lead to the improvement of occupational mental health and wage loss due to presenteeism.

This study has some methodological weaknesses, which are the limitations of this study; there are two major limitations. One is that the saturated models do not allow for comparisons between models. Hence, our model could not be compared with other models, e.g., “SA → insomnia → CCFs → presenteeism”. The other limitation is that this study was not designed to examine the direction of the influence relationship between the parameters because of the cross-sectional study design. Hence, we could not determine the causal relationships between the variables.

This study has several other limitations. First, this study was conducted in Japan, where all the participants were recruited. Hence, the generalizability of these results to other communities may be limited. Second, all the workers were adults, which may prevent the generalizability of these findings to children or adolescents. Third, the study evaluated both participants who were receiving and were not receiving psychiatric treatment and did not differentiate between these groups. Hence, the heterogeneity of the sample may prevent the generalization of the results to mentally healthy or unhealthy individuals. Fourth, the influence of medication at the time of assessment was not controlled for, which may prevent the generalization of these findings to individuals who have not taken any medicine. Fifth, we used self-reported assessments. Although depressive symptoms could influence insomnia, SA, CCFs, and presenteeism, we did not evaluate depressive symptoms in this study. Finally, this study used the participants’ self-assessment to evaluate insomnia, SA, CCFs, and presenteeism. Hence, memory bias may affect those scores. In future investigations, objective assessments (e.g., polysomnography, neurocognitive test, and functional magnetic resonance imaging) rather than self-assessment should be used to evaluate these factors because insomnia, presenteeism, and cognitive dysfunction could very well be measured more equitably. Using objective measures would enable us to explore the common biological factors among insomnia, SA, cognitive dysfunction, and presenteeism.

## 5. Conclusions

The mediating roles of CCFs on the relationship between insomnia, SA, and presenteeism in adult workers were described. To evaluate the presenteeism associated with insomnia and SA adequately, it may be useful to assess the mediating roles of CCFs. In the future, it may be necessary to develop interventions that directly target CCFs to improve occupational mental health.

## Figures and Tables

**Table 1:** Demographic data for 471 study participants.

Characteristic or Measure	Mean (*SD*)	*n* (%)
Age (*n* = 470)	41.0 (11.9)	
Sex (Female) (*n* = 470)		259 (55.1)
Married (*n* = 467)		298 (63.8)
Education (years) (*n* = 471)	14.7 (1.8)	
Psychiatric history (*n* = 471)		53 (11.3)
Current psychiatric treatment (*n* = 466)		19 (4.1)
Drinking (*n* = 471)		310 (65.8)
Smoking (*n* = 471)		96 (20.4)
AIS-J (*n* = 471)	4.2 (3.4)	
STAI-Y state (*n* = 471)	41.6 (9.5)	
COBRA (*n* = 471)	8.4 (6.5)	
WLQ (*n* = 471)	0.04 (0.04)	

AIS-J, Athens Insomnia Scale, Japanese version; COBRA, Cognitive Complaints in Bipolar Disorder Rating Assessment; SD, standard deviation; STAI-Y, State-Trait Anxiety Inventory (Form Y); WLQ, Work Limitations Questionnaire.

**Table 2:** Pearson correlation coefficients (*r*) for 471 study participants.

	AIS-J	STAI-Y State	COBRA
AIS-J	-		
STAI-Y state	0.41 ***	-	
COBRA	0.40 ***	0.29 ***	-
WLQ work productivity loss	0.39 ***	0.35 ***	0.45 ***

*** *p* < 0.001. AIS-J, Athens Insomnia Scale, Japanese version; COBRA, Cognitive Complaints in Bipolar Disorder Rating Assessment; STAI-Y, State-Trait Anxiety Inventory (Form Y); WLQ, Work Limitations Questionnaire.

**Table 3:** Multiple regression analysis for 460 subjects.

	WLQ F (11, 448) = 19.69, *p* < 0.0001		COBRA F (10, 449) = 11.83, *p* < 0.0001	
Independent Variables	*β*	VIF	*β*	VIF
Age	−0.09 *	1.39	0.08	1.39
Sex (Male:1, Female:2)	−0.06	1.25	−0.00	1.25
Married (No:1, Yes:2)	−0.09 *	1.19	−0.10 *	1.17
Education (years)	−0.10 *	1.42	0.02	1.42
Psychiatric history (No:1, Yes:2)	−0.03	1.32	0.06	1.32
Current psychiatric treatment (No:1, Yes:2)	0.08	1.32	0.07	1.32
Drinking (No:1, Yes:2)	−0.06	1.17	0.07	1.16
Smoking (No:1, Yes:2)	−0.09 *	1.12	−0.07	1.11
AIS-J	0.21 ***	1.39	0.32 ***	1.26
STAI-Y state	0.15 **	1.29	0.14 **	1.27
COBRA	0.29 ***	1.26		
Adjusted *R*^2^	0.31		0.19	

* *p* < 0.05, ** *p* < 0.01, *** *p* < 0.001. VIF, variance inflation factor; *β*, standardized regression coefficient; *R*^2^, coefficient of determination; AIS-J, Athens Insomnia Scale, Japanese version; COBRA, Cognitive Complaints in Bipolar Disorder Rating Assessment; STAI-Y, State-Trait Anxiety Inventory (Form Y); WLQ, Work Limitations Questionnaire.

**Table 4:** Standardized path coefficients of path analysis for 471 study participants.

	Direct Effect to		
From	SA	CCFs	Presenteeism
Insomnia	0.410 ***	0.335 ***	0.191 ***
SA		0.154 **	0.181 ***
CCFs			0.321 ***
	**Indirect Effect to**		
	SA	CCFs	Presenteeism
Insomnia		0.063 ** (via SA)	0.074 *** (via SA)
			0.020 ** (via SA and CCFs)
			0.107 *** (via CCFs)
SA			0.049 ** (via CCFs)
	**Total Indirect Effect**		
Insomnia		0.063 **	0.202 ***
SA			0.049 **
	**Total Effect to**		
	SA	CCFs	Presenteeism
Insomnia	0.410 ***	0.398 ***	0.393 ***
SA		0.154 **	0.230 ***
CCFs			0.321 ***

** *p* < 0.01, *** *p* < 0.001. CCFs, complaints of cognitive functions; SA, state anxiety.

## Data Availability

The datasets used and/or analyzed during the current study are available from the corresponding author upon reasonable request.
